# Retention Behavior of Polyethylene Glycol and Its Influence on Protein Elution on Hydrophobic Interaction Chromatography Media

**DOI:** 10.1007/s10337-018-3635-9

**Published:** 2018-11-07

**Authors:** Wojciech Kazimierz Marek, Wojciech Piątkowski, Dorota Antos

**Affiliations:** Faculty of Chemistry, Rzeszow University of Technology, al. Powstańców Warszawy 12, 35-959 Rzeszow, Poland

**Keywords:** Competitive adsorption, HIC, IgG, Polyethylene glycol, Protein separation

## Abstract

The retention behavior of polyethylene glycol (PEG) on different types of hydrophobic interaction chromatography (HIC) resins containing butyl, octyl, and phenyl ligands was analyzed. An incomplete elution or splitting of the polymer peak into two parts was observed, where the first one was eluted at the dead time of the column, whereas the second one was strongly retained. The phenomenon was attributed to conformation changes of the polymer upon its adsorption on hydrophobic surface. The effect enhanced with increasing molecular weight of the polymer and hydrophobicity of the HIC media. Addition of PEG to the mobile phase reduced binding of proteins to HIC resins, which was demonstrated with two model systems: lysozyme (LYZ) and immunoglobulin G (IgG), and their mixtures. In case of LYZ, the presence of PEG caused reduction in the protein retention, whereas for IgG—a decrease in efficiency of the protein capture. The effect depended on the adsorption pattern of PEG; it was pronounced in the systems in which conformational changes of the polymer were suggested to occur.

## Introduction

Production of proteins on an industrial scale is realized in fermentation processes, which output the target product contaminated with specific impurities present in the culture medium and coming from biological material. Therefore, fermentation has to be followed by several downstream operations, where the protein can be isolated out of undesired side components to obtain the final product with high purity and preserved biological activity [1–3]. To satisfy these requirements, a series of different downstream operations is employed, in which precipitation or aqueous two-phase extraction (ATPE) are often used in early purification stages, and followed by chromatographic separations. Both precipitation and ATPE are performed in aqueous solutions, which often contains polyethylene glycol (PEG) in addition to inorganic salts [3–6]. PEG is also frequently used as an additive in other downstream operations to prevent protein denaturation [7]. Therefore, PEG residues can be present in the solvent environment of the protein processed in subsequent chromatographic purification steps. The effect of adding PEG to the mobile phase in ion-exchange (IEX) chromatography has been studied by Lu et al. [7] and Gagnon et al. [8], who proved that PEG can be used to improve the separation selectivity in IEX purifications.

The influence of PEG on the course of protein elution from HIC columns was reported by Hassl and Aspöck [9], who examined purification of hen IgY antibodies from egg yolk, and by Lu et al. [7], who analyzed retention of hemoglobin (Hb). Altering the PEG concentration allowed reducing the protein retention and significant improvement in the protein recovery. The authors suggested the occurrence of competitive adsorption between PEG and the protein as a reason of the observed effects. The possibility of PEG adsorption on HIC columns was confirmed by Werner et al. [10], who measured adsorption isotherms of PEG in aqueous solution of electrolytes. Since PEG molecule exhibits certain hydrophobicity, it can be expected that it is involved in hydrophobic interaction mechanism similar to that underlying adsorption of proteins in HIC systems [11].

In this study, to gain a better insight into adsorption mechanism of PEG on HIC media, we analysed the retention behaviour of two PEG polymers that differed in molecular weight on resins containing butyl, phenyl or octyl ligands. We also examined the influence of PEG on adsorption of proteins in HIC. We selected two model proteins for the study, which exhibited different adsorption patterns on HIC resins, i.e., immunoglobulin G (IgG) and lysozyme (LYZ). The solvent environment selected for the experiments in terms of the concentration of kosmotropic salt and PEG was similar to that typically used in ATPE purification of monoclonal antibodies (IgG) from CHO supernatants [3, 12, 13]. Therefore, the study was also aimed at evaluation of possible conditions and operating variables for capture of IgG with HIC resins as a subsequent step following ATPE.

## Experimental

### Equipment and Materials

The experiments of protein elution were performed on an Äkta purifier station (GE Healthcare, Uppsala, Sweden) with an UV detector. The device was operated by a computer with UNICORN software (GE Healthcare). Elution of PEG was performed using chromatograph LaChrom (Merck, Darmstadt, Germany) equipped with RI Detector L-7490.

The analysis of the molecular weight distribution was performed by gel permeation chromatography (GPC) on PSS GRAM columns (G + 100 + 1000 + 10,000 Å, PSS Polymer Standards Service, Mainz, Germany).

PEG polymers with average molecular weight of 3.35 kDa (PEG-3.35) and 1 kDa (PEG-1) were purchased from Sigma-Aldrich (Poznań, Poland). Two model proteins: lysozyme from chicken egg white (LYZ) and bovine serum immunoglobulin G (IgG) were also purchased from Sigma-Aldrich. All solvents and inorganic salts (analytical grade) were purchased from POCH (Gliwice, Poland).

Three HiTrap 1 mL columns, pre-packed with different Sepharose-based HIC resins with phenyl (*Phe*), butyl (*But*), and octyl (*Oct*) ligands (GE Healthcare) were used; the average pore size of the resin particles was 30 nm [1]. The ligand density characteristics are presented in Table 1. The dimensions of all columns were: 0.7 × 2.5 cm for the ID and the column length, respectively. The voidage of the columns was practically the same, i.e., 95%, which correspond to CV = 0.91 mL (a column volume) for each column. The latter was determined from the retention time of inert peaks (sodium chloride).

**Table 1:** Characteristics of the resins according to the manufacturer information (HiTrap HIC Selection Kit, Data file 18-1143-21 AD, 08/2014)

Resin type	Abbreviation	Ligand density (µmol mL^−1^)
Phenyl sepharose HP	*Phe*	25
Butyl sepharose 4 fast flow	*But*	40
Octyl sepharose 4 fast flow	*Oct*	5

### Procedures

#### Preparation of Mobile Phase

A 50 mM sodium phosphate buffer (PB), pH 7 was prepared as the elution buffer. The salt buffer was an ammonium sulfate (AS) solution with the salt concentration of 1.7M dissolved in PB at pH 7. The mobile phase was a mixture of the salt buffer, PB, and PEG-3.35 or PEG-1 with different concentration.

#### Characterization of Molecular Weight Distribution of PEG Polymers

The molecular weight (MW) distribution of PEG-3.35 and PEG-1 was analyzed using GPC. Three GPC columns, *L* = 300 mm, ID = 8 mm, were assembled in series. Tetrahydrofuran (THF) was used as the mobile phase, with a flow rate (*Q*) of 1 mL min^−1^. The injection conditions were: injection volume (*V*_inj_) 0.1 mL; sample concentration (*C*_PEG,inj_) 5 mg mL^−1^ in THF. The elution profiles were recorded using the RI detector.

#### Elution of PEG and Proteins on HIC Columns

The polymers PEG-3.35 and PEG-1 were dissolved in the mobile phase that contained AS salt, and injected into the columns with the injection volume of 0.1 mL, and the sample concentration *C*_PEG,inj_ = 1–5 mg mL^−1^. The salt content in the mobile phase (*C*_salt_) was increased in subsequent isocratic experiments up to 1.28 M AS. The mobile phase flow rate was *Q* = 0.5 mL min^−1^. The PEG peaks were recoded using RI detector. After each PEG elution experiment, the column was washed with PB free of salt (*C*_salt_ = 0).

LYZ samples were dissolved in the mobile phase that contained AS salt, with and without the addition of the polymer. The injection volume was *V*_inj_ = 0.1 mL, the sample concentration *C*_LYZ,inj_ = 1 mg mL^−1^. The mobile phase composition was changed within the range *C*_salt_ = 0–1.28 M AS. PEG-3.35 or PEG-1 was added into the mobile phase to obtain the polymer concentration within the range *C*_PEG_ = 2–15 mg mL^−1^. The mobile phase flowrate was *Q* = 1 mL min^−1^. The protein peaks were recorded by the UV detector at 280 nm wavelength, and converted into the concentration units using a detector calibration factor.

The operating conditions for IgG were the same as those for LYZ, but the sample concentration was *C*_IgG,inj_ = 0.5 mg mL^−1^. In the case of IgG, only a part of the protein injected was eluted from the column. Therefore, after each protein injection, the same sample was eluted through the detector cell bypassing the column. The calibration factor was calculated for the UV profile of the IgG bypass peak, and used to convert the protein profile at the column outlet (column peak) into the concentration units. The mass of the protein captured on the column was determined by comparing the integrals for the bypass and column peaks.

## Results and Discussion

### Identification of the MW Distribution of PEG-3.35 and PEG-1

The molecular mass of PEG, i.e., 1 kDa and 3.35 kDa, was selected based on the literature reports concerning separation of proteins (e.g. monoclonal antibodies) by extraction in aqueous two-phase systems (ATPE) [4]. As mentioned above (“Introduction”), in this work we aimed at investigation of the influence of PEG on the HIC process subsequent to ATPE. Furthermore, the use of a higher molecular weight of the polymer (above 4000 Da) may involve undesirable effects caused by protein precipitation inside the HIC column.

**Fig. 1:**
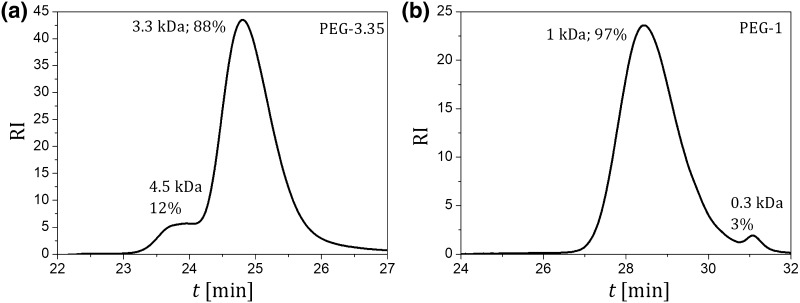
Typical results of the MW distribution analysis by GPC. **a** PEG-3.35 kDa; **b** PEG-1 kDa. *V*_inj_ = 0.1 mL, *C*_PEG,inj_ = 5 mg mL^−1^, *Q* = 1 mL min^−1^

The molecular weight (MW) distribution of PEG was determined using the GPC analysis (“Characterization of molecular weight distribution of PEG polymers”). The results of analysis are presented in Fig. 1.

It was found that for both PEG-3.35 and PEG-1 the MW distribution is relatively uniform. In the case of PEG-3.35, 88% of the molecules were characterized by the average MW of 3.3 kDa, whereas for PEG-1, 97% of molecules had the average MW about 1 kDa.

### Retention Behavior of PEG-3.35 and PEG-1 on Different HIC Media

**Fig. 2:**
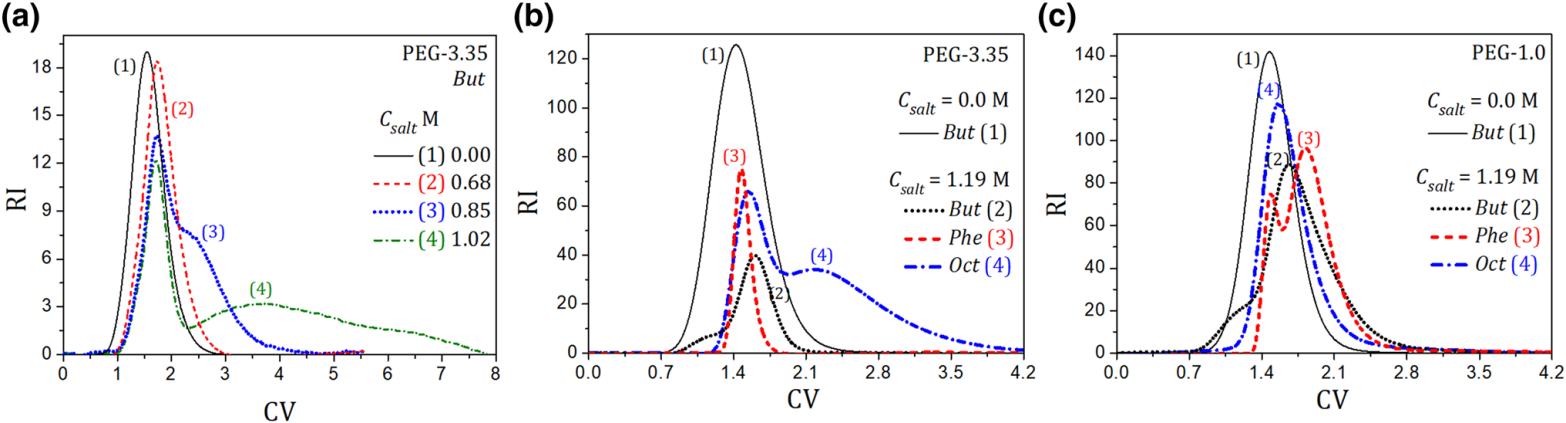
Illustration of the retention pattern of PEG on HIC columns; *V*_inj_ = 0.1 mL, *Q* = 0.5 mL min^−1^. **a** PEG-3.35 on the *But* column at different salt concentration, *C*_PEG,inj_ = 1.0 mg mL^−1^; **b** PEG-3.35 and **c** PEG-1 on the *Phe, But*, and *Oct* columns, *C*_PEG,inj_ = 5.0 mg mL^−1^. For **b** a partial elution of the polymer can be observed (dash-dotted black and dashed red lines). Reproducibility error of the peaks ± 5%

The PEG pulses were eluted from different HIC columns packed with *But, Oct*, or *Phe* adsorbents. The mobile phase contained AS salt with different concentration (“Elution of PEG and proteins on HIC columns”). Typical profiles of PEG-3.35 obtained for the *But* column for different AS salt content in the mobile phase are presented in Fig. 2a. Deformations and splitting of band profiles of the polymer can be observed, which enhanced with increasing salt concentration.

The retention of the first part of the peak was weak and independent of the salt concentration, whereas the retention of the second one increased with increasing salt content. At a sufficiently high AS salt concentration in the mobile phase, the sample of PEG-3.35 was not eluted from the column (Fig. 2b). Nevertheless, binding of PEG-3.35 was reversible; it could be easily desorbed by salt-free solutions. This phenomenon cannot be attributed to heterogeneity of the polymer. As discussed in “Characterization of molecular weight distribution of PEG polymers”, the MW distribution of the polymer was uniform, and most of its molecules had MW equal to 3.3 kDa. The effect also did not depend on the PEG concentration within the range of the column loadings used. Because of dilution of the PEG peaks, effects arising from the viscosity difference between the sample and the mobile phase were negligible. Moreover, because of a large pore size of the matrix particles (about 30 nm) the contribution of the size exclusion effect to the peak splitting phenomenon could also be excluded.

Since the adsorption of PEG and the peak splitting phenomenon occurred only at a high salt concentration, for which hydrophobic interactions dominated, the contribution of hydrophilic interactions could be neglected. Furthermore, PEG was not retained and peak splitting was not observed for a very low ionic strength of the mobile phase, which also excluded the possibility of hydrophilic interactions between the polymer and the matrix.

The retention pattern of PEG was very similar to that observed for unstable proteins in HIC, which was characterized by their incomplete elution from the column in isocratic mode [14–19]. That phenomenon arises from protein unfolding on hydrophobic surfaces. It can be anticipated that PEG, due to its hydrophobic nature, can also change its chain conformation upon adsorption on hydrophobic media. The two-peak elution may indicate existence of two different conformational forms of the polymer, among which only one bounds to the hydrophobic ligands and determines adsorption on HIC media.

The pulse experiments were repeated for the columns with *Oct* and *Phe* ligands. The results obtained for the *Phe* column were similar to those for the *But* column, whereas for the *Oct* one PEG-3.35 was weakly retained due to lower density of the ligand on the adsorbent surface (see “Equipment and materials”). Typical comparison of the retention behavior of PEG-3.35 on different HIC columns is shown in Fig. 2b, where a partial elution of the polymer from *But* and *Phe* columns can be observed. The enhancement of adsorption on the Phe resin is probably caused by additional polar interactions of the polymer with the phenyl ring. The retention behavior of PEG-1 followed similar pattern (Fig. 2c), however, peak splitting was much less pronounced compared to PEG-3.35, which can be explained by lower hydrophobicity of PEG-1 having a shorter polymer chain. Nevertheless, also in this case the peak deformation was observed, which may indicate ability of PEG-1 to change conformation.

### Retention Behavior of LYZ in the Presence of PEG

#### Influence of the Presence of PEG in the Mobile Phase

**Fig. 3:**
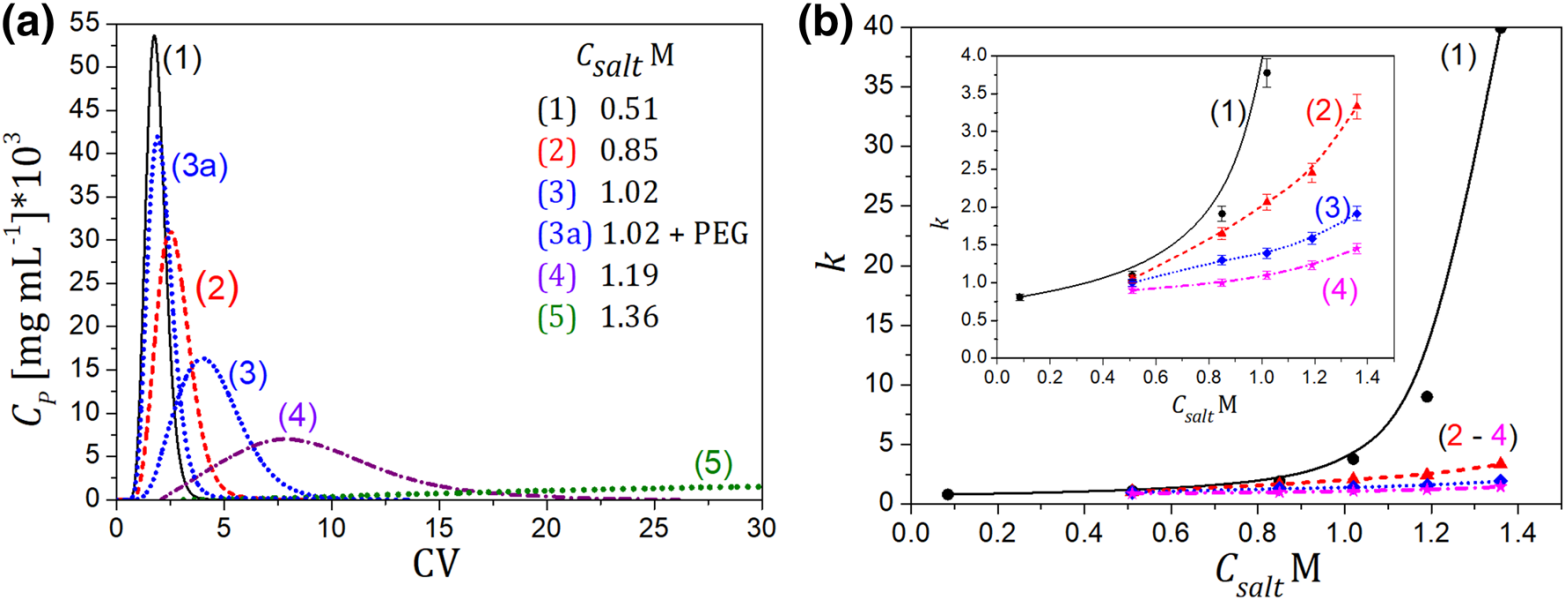
Illustration of the retention behavior of LYZ on the *But* column; *V*_inj_ = 0.1 mL, *C*_LYZ,inj_ = 1 mg mL^−1^, *Q* = 1 mL min^−1^. **a** At different compositions of the mobile phase, curves 1–5—no PEG added, curve 3a—PEG-3.35 added into the mobile phase (*C*_PEG_ = 10 mg mL^−1^), reproducibility error of the peaks ± 5%; **b** changes of the retention factor (*k*) vs the AS salt concentration, at different PEG-3.35 content in the mobile phase, *C*_PEG_: (1) 0; (2) 2; (3) 5; (4) 10 mg mL^−1^. Lines guide the eye

The pulse elution experiments were performed for LYZ at different compositions of the mobile phase in isocratic mode. The AS salt and PEG concentrations were changed within the range: 0–1.19 M AS, and 0–10 mg mL^−1^ PEG-3.35 or PEG-1. The changes of the retention behavior of LYZ vs the mobile phase composition are illustrated in Fig. 3a, where typical elution profiles are shown, and Fig. 3b, where changes of the protein retention factor, *k*, are depicted.

An increase in the salt concentration enhanced the protein retention by promoting hydrophobic interactions of the protein with ligands on the adsorbent surface, while addition of PEG to the mobile phase, even in a small amount, caused the protein retention to reduce significantly.

**Fig. 4:**
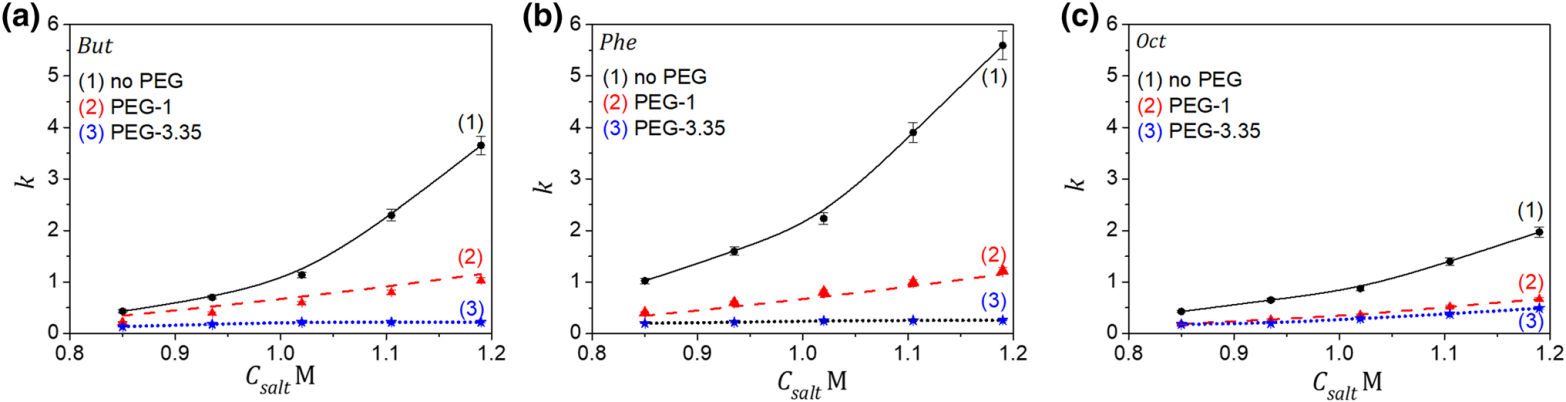
Changes of the retention factor (*k*) of LYZ on different HIC columns vs the AS salt concentration at the absence or presence of PEG in the mobile phase (C_PEG_ = 10 mg mL^−1^), *V*_inj_ = 0.1 mL, *C*_LYZ,inj_ = 1 mg mL^−1^, *Q* = 1 mL min^−1^. **a** *But;* **b** *Phe*; **c** *Oct* columns. Lines guide the eye

In general, the presence of PEG in the protein solutions is known to reduce the solubility of proteins and promote hydrophobic interactions, which is expected to drive protein adsorption. However, it is obvious that the effect of competitive adsorption between the polymer and the protein dominated in the retention mechanism. The presence of PEG-3.35 with the concentration above 2 mg mL^−1^ caused LYZ to elute in flow-through mode regardless of the AS salt concentration. The PEG effect depended on the polymer molecular weight and type of the HIC adsorbent, which was illustrated in Fig. 4.

As expected, the retention changes were the least pronounced for PEG-1 on the *Oct* column, which stemmed from the weakest adsorption of the polymer on the octyl ligands in that column.

#### Influence of the Presence of PEG in the Sample Solvent

**Fig. 5:**
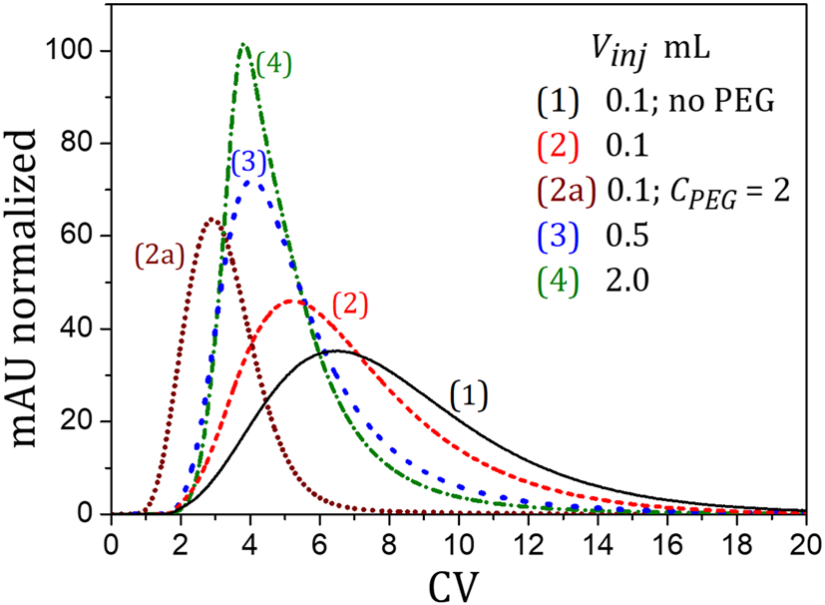
Illustration of the sample solvent effect for LYZ. The salt concentration in the sample and in the mobile phase *C*_salt_ = 1.19 M; lines: (1) no PEG added; (2)–(4) PEG-3.35 only in the sample solvent, *C*_PEG,inj_ = 2 mg mL^−1^; (2a) PEG-3.35 in the sample solvent and in the mobile phase. *C*_PEG_ = *C*_PEG,inj_ =2 mg mL^−1^. *C*_LYZ,inj_ = 1 mg mL^−1^, *Q* = 1 mL min^−1^. Reproducibility error of the peaks ± 5%

The elution of LYZ was also strongly altered when PEG was present in the solvent of the protein sample, but absent from the mobile phase. Because of the competitive adsorption, the protein eluted earlier than in the absence of PEG, which is illustrated in Fig. 5.

As can be observed, the shift in retention enhances with increasing sample volume, when the sample solvent and the protein can interact longer during their migration through the column. Also, for a low injection volume, the interactions weaken due to dilution of the sample solvent in the mobile phase [20].

### Retention Behavior of IgG

**Fig. 6:**
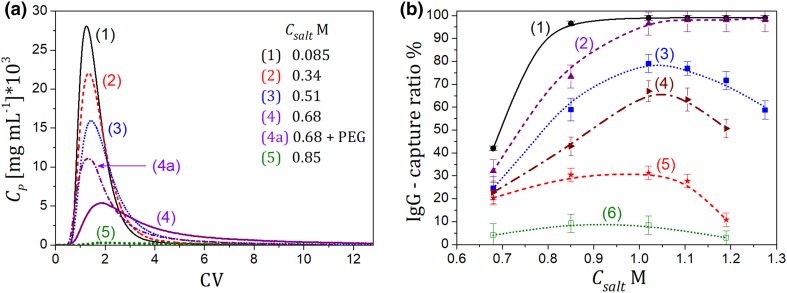
Illustration of the retention behavior of IgG on the *But* column, for different compositions of the mobile phase. **a** Curves (1–5)—no PEG added; curve (4a)—PEG-3.35 added, *C*_PEG_ = 10 mg mL^−1^, reproducibility error of the peaks ± 5%; **b** adsorption behavior of IgG on the *But* column vs the salt concentration at different PEG-3.35 content in the mobile phase, *C*_PEG_: (1) 0; (2) 2; (3) 5; (4) 7; (5) 10; (6) 15 mg mL^−1^. Capture ratio defined by Eq. (1). The following conditions were used: *V*_inj_ = 0.1 mL, *C*_IgG,inj_ = 0.5 mg mL^−1^, *Q* = 1 mL min^−1^. Lines guide the eye

IgG exhibited incomplete elution on all HIC columns, which can be ascribed to unfolding of the protein molecule upon adsorption. Typical band profiles recorded in isocratic elution are shown in Fig. 6. The eluting peak can be assigned to the native form of the protein, which is usually weakly adsorbed, therefore it can be eluted with the mobile phase [14, 15]. The unfolded form of the protein, which is strongly bound to the adsorbent surface, is retained in the column. That phenomenon caused the depletion in the peak area visible in Fig. 6a, which enhanced with increasing the salt concentration. Desorption of the unfolded form could be imposed by reduction in the salt concentration in the mobile phase, when the protein could refold and elute from the column. Such a phenomenon has already been described for different proteins with unstable structure adsorbed on HIC media, also including a monoclonal antibody IgG1 [12–19]. The presence of PEG diminished that effect, which is illustrated in Fig. 6a (peak 4 and 4a).

To quantify the changes in the adsorption strength for IgG in a function of the mobile phase composition and the adsorbent type, the ratio of the protein mass captured to that eluted from the column was calculated as follows:

1 $${\text{capture}}\;{\text{ratio}}~=~\left( {1 - ~\frac{{{m_{{\text{p,e}}}}}}{{{m_{{\text{p,inj}}}}}}} \right)~100\% ,$$

where *m*_p,e_ is the protein mass detected in the mobile phase at the column outlet (in eluate) calculated from the UV detector response (at 280 nm); *m*_p,inj_ is the protein mass in the sample.

The changes in the capture ratio of IgG on the *But* column in the presence of PEG-3.35 are illustrated in Fig. 6b. Adsorption of the protein enhanced with increasing the salt concentration in the mobile phase in the absence of the polymer. The salt dependency of the retention maintained that trend for the PEG concentration up to 2 mg mL^−1^. At a higher content of the polymer, and at the salt concentration above 1 M of AS, the effect of the PEG presence dominated that of the salt, due to the enhancement of competitive adsorption between PEG-3.35 and the protein. The amount of the protein captured on the column reached a maximum at about 1 M AS, and above that concentration it visibly decreased, i.e., the area for the protein peak assigned to its native form increased. Therefore, further addition of the salt to the mobile phase reduced the efficiency of the protein capture, which was against to typical retention trends of proteins on HIC media. It can be deduced that the presence of PEG in the adsorbed phase prevented IgG from unfolding. This probably stems from steric hindrances for the protein mass transfer caused by congestion of pore free space by the adsorbed PEG molecules. A similar effect was reported in other studies [18, 19], where the phenomenon of protein unfolding was shown to diminish with increasing the column mass loading.

**Fig. 7:**
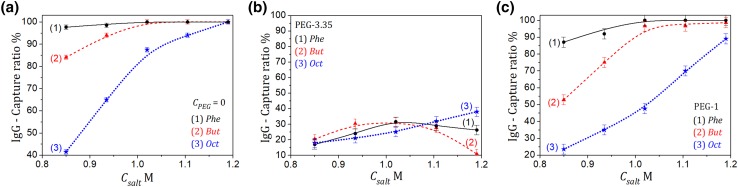
Adsorption behavior of IgG on different HIC columns vs the salt concentration in the absence and presence of PEG in the mobile phase. **a** no PEG added; **b** with PEG-3.35, *C*_PEG_ = 10 mg mL^−1^; **c** with PEG-1, *C*_PEG_ = 10 mg mL^−1^. *V*_inj_ = 0.1 mL, *C*_IgG,inj_ = 0.5 mg mL^−1^, *Q* = 1 mL min^−1^. Lines guide the eye

The effect described above was also present during IgG elution on the *Phe* column, whereas for the *Oct* one it was less pronounced due to weak adsorption of the polymer on that column (Fig. 7). The adsorption behavior also depended on the polymer molecular weight; as expected, the weakest influence of PEG on the adsorption behavior of IgG was observed for weaker adsorbed PEG-1 (Fig. 7c).

The effect of the sample solvent on the protein elution followed similar trend as that reported for LYZ in “Influence of the presence of PEG in the sample solvent”; adsorption strength of the protein reduced with increasing the sample volume.

### Elution of the Protein Mixtures

**Fig. 8:**
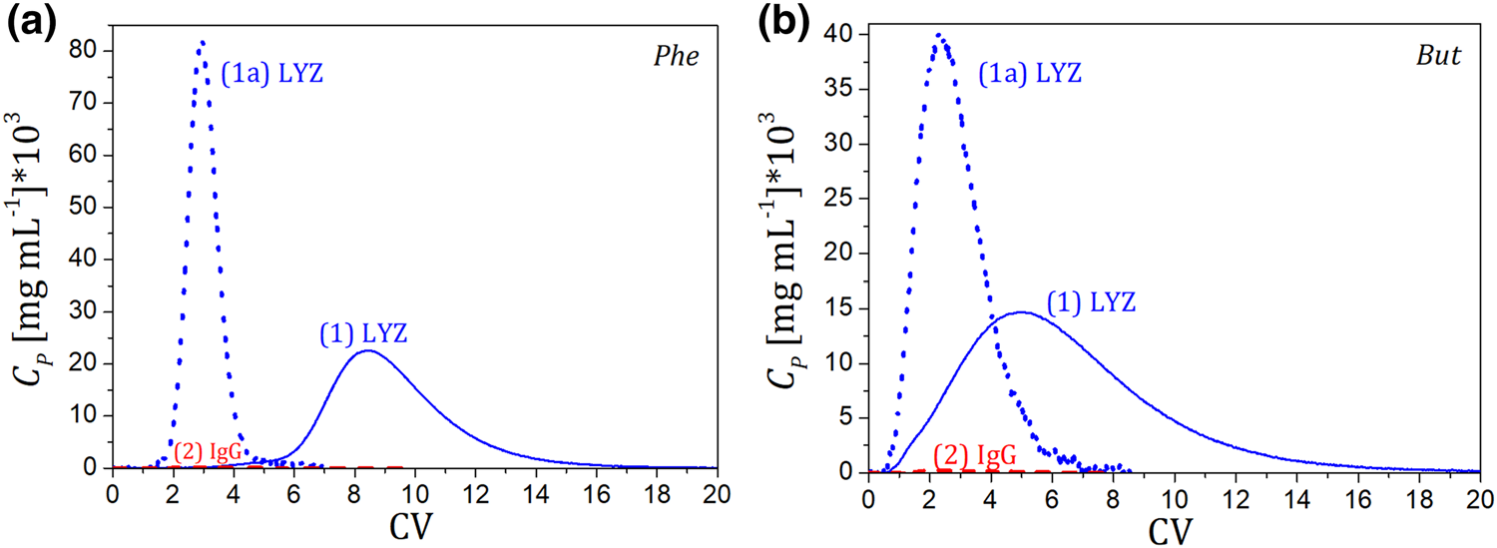
Separation of IgG and LYZ **a** on the *Phe* column, **b** on the *But* column. Lines: (1) LYZ eluted in the absence of PEG in the mobile phase; (1a) and (2) LYZ and IgG, respectively, injected in the presence of PEG-1 in the mobile phase (*C*_PEG_ = 10 mg mL^−1^) at which IgG is retained on the column (the capture ratio was ~ 98%). *V*_inj_ = 0.1 mL, *C*_LYZ,inj_ = 1.0 mg mL^−1^, *C*_IgG,inj_ = 0.5 mg mL^−1^, *Q* = 1 mL min^−1^

The retention behavior of both proteins LYZ and IgG markedly differed; therefore, the effect of the PEG presence on the adsorption of proteins was also different. As described above, in the case of LYZ, even a small addition of PEG to the mobile phase diminished the protein adsorption on HIC columns, whereas for IgG the adsorption strength could be tuned by the molecular weight of PEG and its concentration in the mobile phase. This difference might be potentially exploited to improve the selectivity of separations of IgG from protein impurities on HIC media. An example of that approach is provided in Fig. 8, where the results of chromatographic elution of IgG in a binary mixture is illustrated.

The PEG and salt concentrations were selected in such a way that LYZ was eluted in flow-through mode, while IgG was completely retained in the column at the same mobile phase composition. The same efficiency of the separation can be achieved using PEG-3.35 instead of PEG-1; however, the polymer concentration in the mobile phase has to be reduced fivefold (data not shown).

As reported above, in the presence of PEG, an increase of the salt concentration in the mobile phase may result in reduction of the protein adsorption strength instead of its enhancement, therefore, both salt and PEG concentrations should be properly optimized to minimize yield loss of the target protein in HIC purifications.

The operating window for the PEG concentration, which allowed the capture of IgG on the HIC columns, was within the range 2–10 mg mL^−1^, at the AS salt concentration 1–1.2 M (depending on the polymer MW). Such a solvent environment is similar to that occurring in a bottom salt phase in ATPE purifications of monoclonal antibodies from CHO supernatants [4, 12, 13]. It can be concluded that a post-extraction salt phase could possibly be processed in HIC column without much investment in the solvent exchange operation.

Finally, the stability of the proteins adsorbed on the investigated HIC media in the presence of PEG and AS salt was analyzed using the DSF method (differential scanning fluorimetry) according to the procedure described in a previous work [19]. In all cases the fluorescence curves and the unfolding characteristic points for the native and adsorbed proteins remained the same (data not shown), which proved that the protein stability in the adsorbed phase was preserved. This could be expected, due to the mild conditions of the HIC process and the properties of PEG, which is often added to solutions of proteins to stabilize their structure.

## Conclusions

The retention behaviour of PEG-3.35 kDa and PEG-1 kDa on different HIC columns has been analysed. In case of *But* and *Phe* type of HIC media, the adsorption of PEG was strong and increased with the salt concentration. Moreover, partial elution of the polymer on the column was observed, which was attributed to a change of the polymer chain conformation upon adsorption. The phenomenon was active for both PEG-3.35 and PEG-1; however, it was more pronounced for the polymer with the higher molecular weight.

The experiments performed indicated that the influence of PEG on the protein retention can be explained by competitive adsorption between the protein and the polymer. In case of both model proteins (LYZ and IgG), the presence of PEG in the mobile phase caused a decrease in their adsorption strength; however, this effect was markedly stronger for LYZ. Therefore, the content of PEG in the mobile phase could be altered to amend the selectivity of the protein separation. Thus, the residual presence of PEG in the protein mixtures, which are provided from preliminary purification stages, may be treated as a mobile phase modifier in the HIC process. Its content can be optimized to improve yield and productivity of the operation.

Since the solvent environment of chromatographic experiments performed in this study was similar to that occurring in ATPE separations of IgG, the results of the study may provide hints for design of a coupled process of ATPE and HIC, e.g. for antibody purifications. Nevertheless, the choice of the process conditions depends on the type of the target product and the character of impurities to be separated.
